# Mechanical performance of porous biomimetic intervertebral body fusion devices: an in vitro biomechanical study

**DOI:** 10.1186/s13018-023-03556-4

**Published:** 2023-01-30

**Authors:** Fon-Yih Tsuang, Ming-Jun Li, Po-Han Chu, Nien-Ti Tsou, Jui-Sheng Sun

**Affiliations:** 1 Division of Neurosurgery, Department of Surgery, National Taiwan University Hospital, No.7, Chung-Shan South Rd., Taipei, 10002 Taiwan, ROC; 2 Department of Materials Science and Engineering, National Yang Ming Chiao Tung University, Hsinchu, Taiwan, ROC; 3 Research & Development, Ingrowth Biotech. Co., Ltd., 1F, No. 57, Luke 2nd Road, Luzhu District, Kaohsiung Science Park, Kaohsiung, 82151 Taiwan, ROC; 4 Trauma and Emergency Center, China Medical University Hospital, No.2, Xueshi Rd., North Dist., Taichung City, 404018 Taiwan, ROC; 5 Department of Orthopedic Surgery, College of Medicine, China Medical University, No. 2, Yu-Der Rd, Taichung City, 40447 Taiwan, ROC; 6 Department of Orthopedic Surgery, National Taiwan University Hospital, No.7, Chung-Shan South Rd., Taipei, 10002 Taiwan, ROC

**Keywords:** Biomimetic, Cervical intervertebral body fusion device, Mechanical test, Finite element

## Abstract

**Background:**

Degenerative disc disease is one of the most common ailments severely affecting the quality of life in elderly population. Cervical intervertebral body fusion devices are utilized to provide stability after surgical intervention for cervical pathology. In this study, we design a biomimetic porous spinal cage, and perform mechanical simulations to study its performances following American Society for Testing and Materials International (ASTM) standards before manufacturing to improve design process and decrease cost and consumption of material.

**Methods:**

The biomimetic porous Ti-6Al-4 V interbody fusion devices were manufactured by selective laser melting (laser powder bed fusion: LPBF in ISO/ASTM 52900 standard) and subsequently post-processed by using hot isostatic pressing (HIP). Chemical composition, microstructure and the surface morphology were studied. Finite element analysis and in vitro biomechanical test were performed.

**Findings:**

The post heat treatment can optimize its mechanical properties, as the stiffness of the cage decreases to reduce the stress shielding effect between two instrumented bodies. After the HIP treatment, the ductility and the fatigue performance are substantially improved. The use of HIP post-processing can be a necessity to improve the physical properties of customized additive manufacturing processed implants.

**Interpretation:**

In conclusion, we have successfully designed a biomimetic porous intervertebral device. HIP post-treatment can improve the bulk material properties, optimize the device with reduced stiffness, decreased stress shielding effect, while still provide appropriate space for bone growth.

**Clinical significance:**

The biomechanical performance of 3-D printed biomimetic porous intervertebral device can be optimized. The ductility and the fatigue performance were substantially improved, the simultaneously decreased stiffness reduces the stress shielding effect between two instrumented bodies; while the biomimetic porous structures provide appropriate space for bone growth, which is important in the patients with osteoporosis.

**Supplementary Information:**

The online version contains supplementary material available at 10.1186/s13018-023-03556-4.

## Introduction

Cervical spondylosis is a common clinical disease in the middle-aged and elderly populations with a trend of getting younger [1]. Anterior cervical discectomy and fusion (ACDF) can provide stability during vertebral body movement and is one of the most widely accepted procedures performed for both single and multi-level cervical disc diseases [2, 3]. Due to the associated disadvantages of autograft, such as bone absorption, pseudarthrosis, and donor site morbidity been concerned for its clinical practice; the use of a cervical interbody fusion cage is recommended. By providing strong anterior support for solid fixation and fusion, its architecture can obviate the need for bone grafting [4]; but, inappropriate implants may also cause the instability of the fusing spine and damage to the endplate or neural structure [5]. The most commonly used fusion cage materials in current clinical practice are titanium (Ti) alloys and polyetheretherketone (PEEK) [6, 7].

Three-dimensional (3-D) printing refers to the process of fabricating fusion by way of high-precision, rapid-fabrication and customized-production [8]. Despite the increasing application of 3D printing in bone tissue engineering, there are few investigations into the spinal interbody fusion device; current evidence of its use is still limited to animal studies and only few human case series [9]. In the recent study, 3-D printed porous titanium cervical implants demonstrated faster consolidation, better stability and similar fusion rate when compared with PEEK or with autograft [10].

The biomechanical properties of the implant are essential to its stability [11]. According to Wolfe’s law, the structure of the bones is suitable for resisting any force acting on the bones, and the bone mass is reduced in response to low stress [12]. A structure with lower stiffness should theoretically maintain a certain degree of movement and reduce the stress on the facet joints and intervertebral discs in adjacent positions [13]. The lower the stiffness of the implant, the smaller the stress on the endplate, the lower possibility of occurrence of microfractures, osteolysis, or cage subsidence. It was measured by Grant et al. that the stiffness of different areas on the endplate exhibited a trend of decreasing from the outside to the center of the endplate [14]. Microfractures occur when the local stress is higher than the limit of the relevant area [14, 15], leading to osteolysis and cage subsidence [14, 15]. A previous study reported that micro-movements greater than 150 μm reduced the interface bonding strength, eventually resulting in implant relaxation [16].

When an implant was engineered with porous-structure, a significant amount of energy was absorbed by the implant instead of being transferred to the surrounding cortical bone. The load-sharing capacity was effectively enhanced and the maximum Equivalent von Mises (EQV) stress was reduced in the adjacent cortical bone [17]. An implant inherent with porosity had less effect on implant displacement, while the inner pore structure in this design could enhance the bone ingrowth. Additive manufactured medical instruments with porous structures provide more flexible design principles by optimizing a design and bring lots of benefits, such as functionally gradient structure for cells to proliferate, lower consumption of materials, and customized strength and stiffness [18, 19].

Additive manufacturing is the currently available manufacturing technology applied to fabricate the scaffold according to the predefined computer aided design (CAD) model [20]. Because different porous shapes will make a device have different failure properties, such as failure direction, which result in the whole process more complicated [21, 22], the whole process from design to estimation is time-consuming and expensive [19]. Finite element methods have lots of application in the biomedical field, such as spinal cage evaluations [23], stent device analysis [24], and dental implant evaluations [25]. To avoid waste of materials and time and increase design efficiency, we adopt finite element methods to perform a mechanical simulation and assess spinal cages by using ASTM standards. In our work, we designed a biomimetic porous spinal cage, performed mechanical simulations to study its biomechanical performances.

## Materials and methods

### Manufacture and heat treatment of the biomimetic porous device

**Fig. 1:**
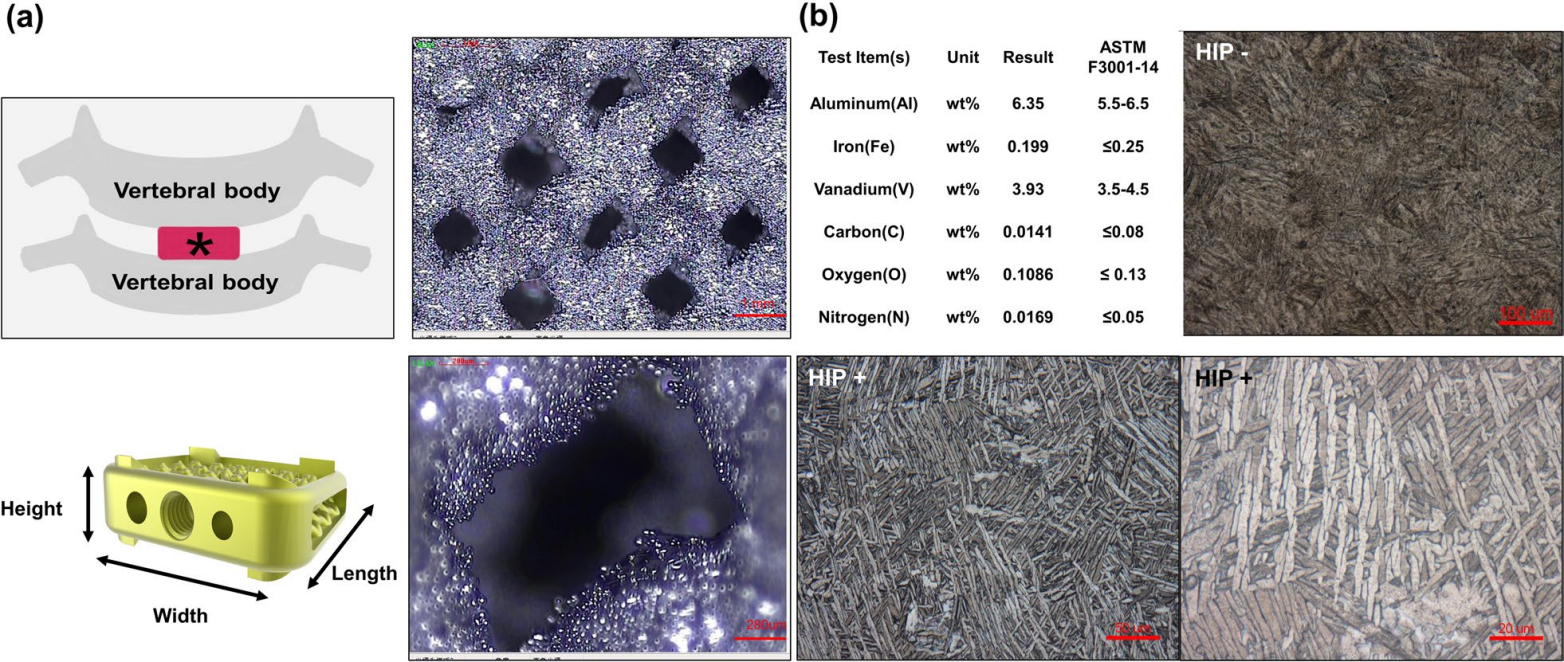
The schematic details, chemical composition and metallography observation of intervertebral fusion device. **a** The schematic details of cervical spine fusion surgery and dimension recorded for intervertebral fusion device. Asterisk: intervertebral fusion device (cage). For the present study, the experimental cage was similar configuration to that of EIT Cervical Cage (Natural Bone Ingrowth with EIT Cellular Titanium^®^; EIT Emerging Implant Technologies GmbH, Tuttlingen, Germany) and was produced using the Renishaw AM 400 system (UK). The representative images of samples from each batch are shown; with the dimensions of 15 mm in width, 9 mm in height, and 13 mm in length. All samples show a similar level of porosity. There are non-melted surface powder particles visible on the sides of the pores which indicates insufficient melting during laser beam movement. The centerline average roughness (Ra) was 10–30 μm, pore size 500-600 μm, and the porosity ratio was 55%. **b** Chemical Composition and Metallography Observation. Metallographic organizations presented such as attached photos. The metallographic structure of the sample showed acicular structure without obvious grain display. After hot isostatic pressing treatment, the acicular structures become wider and less sharp. HIP treatment: − (without hot isostatic pressing); HIP treatment: + (with hot isostatic pressing)

For the present study, the interbody fusion device was a template and evolved from the profile of EIT Cervical Cage (EIT Emerging Implant Technologies GmbH, Tuttlingen, Germany), with identical geometric parameters in cage length, width, and height. The cages were produced using the Renishaw AM 400 system (UK). The interconnected porous structure and surface roughness was integrated into the manufacturing process of the device. The centerline average roughness (Ra) was 10–30 μm, pore size 500-600 μm, and the porosity ratio was 55%. In vitro mechanical tests, including compression, shear, torsional tests, and fatigue test, were performed to evaluate the mechanical specifications and strength of the experimental devices. The representative picture of devices can be seen in Fig. 1. Following selective laser melting (SLM), hot isostatic pressing (HIP) was performed for the samples using a HIP vessel. Samples were hot isostatic pressed at 890 °C (holding time: 120 min; heating rate: 0.5 °C min^−1^). The temperature applied was 890 °C of annealing was to reduce residual stress without significant microstructural changes. The slow cooling can effectively eliminate residual stresses without introducing further ones during cooling. Chemical reaction between the free metallic surface and the atmosphere was avoided by performing the heat treatment in a high pressure (100 MPa) inert gas (argon) atmosphere. Samples were divided into two categories according to the planned heat treatment (with HIP, without HIP) for the following study.

### Characterization of the porous biomimetic Ti6Al4V interbody fusion devices

Two-dimensional measuring instrument (JMT2010, Jingsh Meng Technology Co. ltd, Taichung, Taiwan) was used to study the surface morphology, microstructure and pore morphology within the structure. The micro structural surface characteristics and pore morphology within the structure were studied by scanning electron microscopy (SEM). The porosity and strut volume of the implant were determined using a helium-purged Low Pressure Pycnometer (LPP) (Micromeritics® ASAP 2060 Pycnometer, Accelerated Surface Area and Porosity System, Micromeritics Instruments Corporation, Norcross, GA, USA).

#### Observation of chemical composition, microstructure and metallography

The material compositions of titanium implant manufactured were tested by inductively coupled plasma atomic emission spectroscopy (ICP-AES), which was meet the ASTM F3001-14 (Standard Specification for Additive Manufacturing Titanium-6 Aluminum-4 Vanadium ELI (Extra Low Interstitial with Powder Bed Fusion).

### Evaluation of the mechanical properties of the implants per ASTM F2077

#### Finite element methods

The non-linear elastic–plastic performance of the biomimetic cage was studied using finite element model (ANSYS workbench v.19 software). The Ansys non-linear mechanical solver was used to simulate the structural behavior closely following the physical test conditions. We used Static Structural and Fatigue modules to simulate mechanical test procedures, and follow the ASTM F2077 and ASTM F2677 standards, which could calculate the mechanical performance of the cage, such as fatigue life and subsidence behavior.

##### Mesh convergence tests

Here, tetrahedral meshes were adopted in the current models. This is because the biomimetic porous structures are typically too complicated for hexahedral meshes. The maximum stress was selected as an index for examining the convergence of models with different mesh sizes. When the maximum stress difference between two mesh sizes is less than 10%, the greater mesh size is chosen and used in the current work.

##### Boundary conditions and settings in the finite element models

**Fig. 2:**
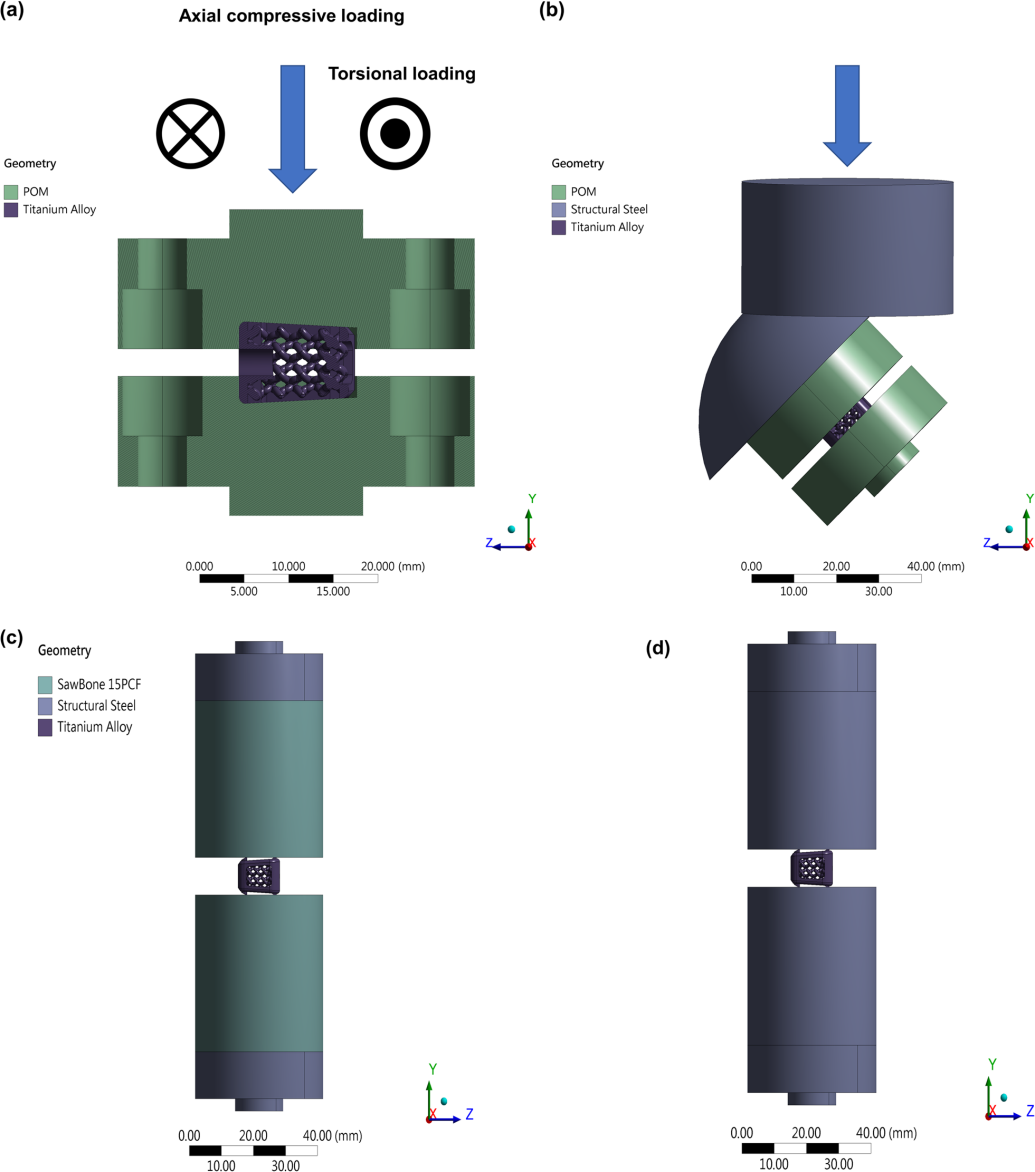
The finite element (FE) model used to perform mechanical tests. **a** The FE boundary conditions for the axial compressive tests and torsional tests. **b** The model to simulate the shear compressive test. **c** The FE boundary conditions for the subsidence tests. **d** The model used to calculate stiffness of the intervertebral body fusion device. Polyoxymethylene: POM

In the current work, the non-linear elastic–plastic behavior of the biomimetic cages was modeled by bi-linear isotropic hardening with elastic modulus *E* = 108.7 GPa and yielding stress *Y* = 849.7 MPa. The hardening model and setting were also used by Ricardo Chávez-Vásconez et al. (2021) [26]. Four types of tests were simulated by finite element (FE) models where the non-linear elastic–plastic behavior was modeled by bi-linear isotropic hardening with elastic modulus *E* = 108.7 GPa and yielding stress *Y* = 849.7 MPa. (1) Axial compressive tests, where the forces of 2000, 2400, and 2600 N along the -y direction were applied on the surface marked in red while the surface marked in blue was fixed in all directions as shown in Fig. 2a. (2) Torsional tests, where the torque of 2 N-m around the y axis was applied on the surface marked in red while the surface marked in blue was fixed in all directions as shown in Fig. 2a. (3) Shear compressive tests, where the entire model was rotated and steel loaders were added as shown in Fig. 2b. The forces of 1400 and 1875 N along the -y direction were applied on the surface marked in red while the surface marked in blue was fixed in all directions. (4) Subsidence tests, where sawbones and steel loaders were added to the model. A downward displacement was applied on the surface marked in red while the surface marked in blue was fixed in all directions as shown in Fig. 2c. Note that the simulation stopped when the curve of force and displacement reached the yield platform.

In the dynamic fatigue test, the model used to perform the axial compressive test and torsional test are the same as illustrated in Fig. 2a; the model used in the shear test has a tilt angle (a sagittal inclination of 45° test setup) to simulate the shear effect as shown in Fig. 2b in order to fit experiments in real world. Our dynamic fatigue models are based on the S–N curve and Goodman's mean stress correction theory to simulate dynamic fatigue tests. According to the ASTM F2077 standard, the axial compressive test and shear compressive test are estimated under a 0.1 fatigue ratio. However, the fatigue ratio of the S–N curve we used in our simulation ratio is -1. To compensate for the difference between those two fatigue ratios, we adopt Goodman's mean stress theory. Goodman's mean stress theory has been widely used to address the problem of a different fatigue ratio from metal materials test [27]. Regarding the cage subsidence test, the finite element models are shown in Fig. 2c, d. Following the ASTM F2677 standard, we need to use Eq. (1) to calculate the stiffness of test block ($\mathrm{Kp}$) that is a combination of the stiffness of the system ($\mathrm{Ks}$) and the stiffness of the intervertebral body fusion device ($\mathrm{Kd}$).

1 $$\mathrm{Kp}=\frac{\mathrm{KsKd}}{\mathrm{Kd}-\mathrm{Ks}}$$

According to the ASTM F2077 standard, dynamic fatigue tests are used to analyze the mechanical performance of the cage. Based on our design experience, we aimed to design a cage with a minimum of 2000N in dynamic axial compressive test, minimum 1400N shear compressive test, and 2N-m dynamic torsional test. The materials used in the mechanical simulation and the boundary conditions for dynamic fatigue tests are well defined (Additional file 1: Table S1**)**. Regarding the subsidence test, we applied a displacement until the slope of a curve in a force–displacement graph is close to zero. The test blocks in fatigue tests were made of polyacetal whose ultimate tensile strength is 72 MPa; the ones in the subsidence test were made of Grade 15 polyurethane to simulate vertebral bodies. Both two types of test blocks are defined and suggested by the ASTM F2077 and ASTM F2677 standards respectively. The fatigue material property of titanium alloy is based on the S–N curve that is from Benedetti et al. [28, 29].

#### Real in-vitro mechanical test

By using a material test machine (MTS 370, MTS System Corporation, MN, USA) and MTS torsional load cells (662.18H-05, Axial capacity25 KN, Torsional Capacity250 N-m), failure of the implants was evaluated. Six mechanical tests, including static axial compression, compression-shear, torsion test, dynamic axial compression, compression-shear, and torsion test were performed under the guide FDA per the methods described in ASTM F2077 [3]. Briefly, in each of these tests, devices are fixtured into a load frame and a static or dynamic load or torque is applied. Static tests are run until device or device-fixture interface failure, or test machine limits are reached; while dynamic tests are run until device failure occurs or the device reaches 5 million cycles without failure.

The tested cage was at the dimensions of 15 × 9 × 13 mm (width x height x length). In the compression-shear test, a sagittal inclination of 45° test setup was utilized. A 100-N axial preload was performed before the torsional test; while fully reversed loads were used for dynamic torsion tests as specified in ASTM F2077. For each static test, the mean and standard deviation of stiffness, yield load/torque, and ultimate load/torque were collected. Based on the manufacturer’s description, the failure mode and photographs of failed devices were also documented for each test. Characterizations of yield and ultimate behavior were made based on load vs. displacement plots. For each dynamic test, the following information was collected as reported: number of specimens that achieved the runout load, cyclic load or torque at which the device did not exhibit any failures after reaching 5 million cycles (i.e., the runout load), and the failure load at which a specimen failed prior to reaching 5 million cycles. Tensile testing was performed per ASTM E8/E8M-2016a by using cylindrical dog-bone-shaped specimens built using SLM in their shape (cross-section: 6 mm in diameters).

#### Subsidence testing per ASTM F2267

Subsidence testing, as described in ASTM F2267, is intended to characterize the propensity of an intervertebral body fusion devices (IBFDs) to subside into the vertebral body endplates. Subsidence testing involves placing the intervertebral body fusion devices (IBFDs) between two blocks of “Sawbones” (polyurethane test block, product number: 1522–02, Pacific Research Laboratories Inc., U.S.), which intended to replicate compression properties of trabecular bone, and applying a compressive load. Per ASTM F2267, the relative propensity of a device to subside is quantified by a stiffness measurement, Kp (N/mm), which represents the stiffness of the foam block as it deforms under the loads applied. A higher stiffness measurement is generally expected to represent that a device is more resistant to subsidence into a vertebral body.

### Mechanical testing data analysis

Data for each mechanical performance parameter (static testing: stiffness, yield, and ultimate strength; dynamic testing: runout load; subsidence testing: block stiffness [Kp]) were calculated for each static performance result. The most common failure modes for each test were determined using the photographic documentation provided. Statistical assessment was performed on SPSS 15.0 (IBM Corporation, Armonk, NY). All data are presented as mean ± standard deviation (SD). Statistical significance was defined as p < 0.05.

## Results

### Device design and microstructure

The representative images of samples from each batch are shown in Fig. 1a; with the dimensions of 15 mm in width, 9 mm in height, and 13 mm in length. All samples show a similar level of porosity and the porosity was created exclusively during the stage of the material production process. The character of the pores is analogical for all samples, there are non-melted surface powder particles visible on the sides of the pores. The centerline average roughness (Ra) was 10–30 μm, pore size 500-600 μm, and the porosity ratio was 55%.

### Chemical composition and metallography observation

Metallographic organizations presented such as attached photos at Fig. 1b. The metallographic structure of the sample showed acicular structure display. After hot isostatic pressing treatment, the acicular structures become wider and less sharp.

### Finite element model: axial compressive test

**Fig. 3:**
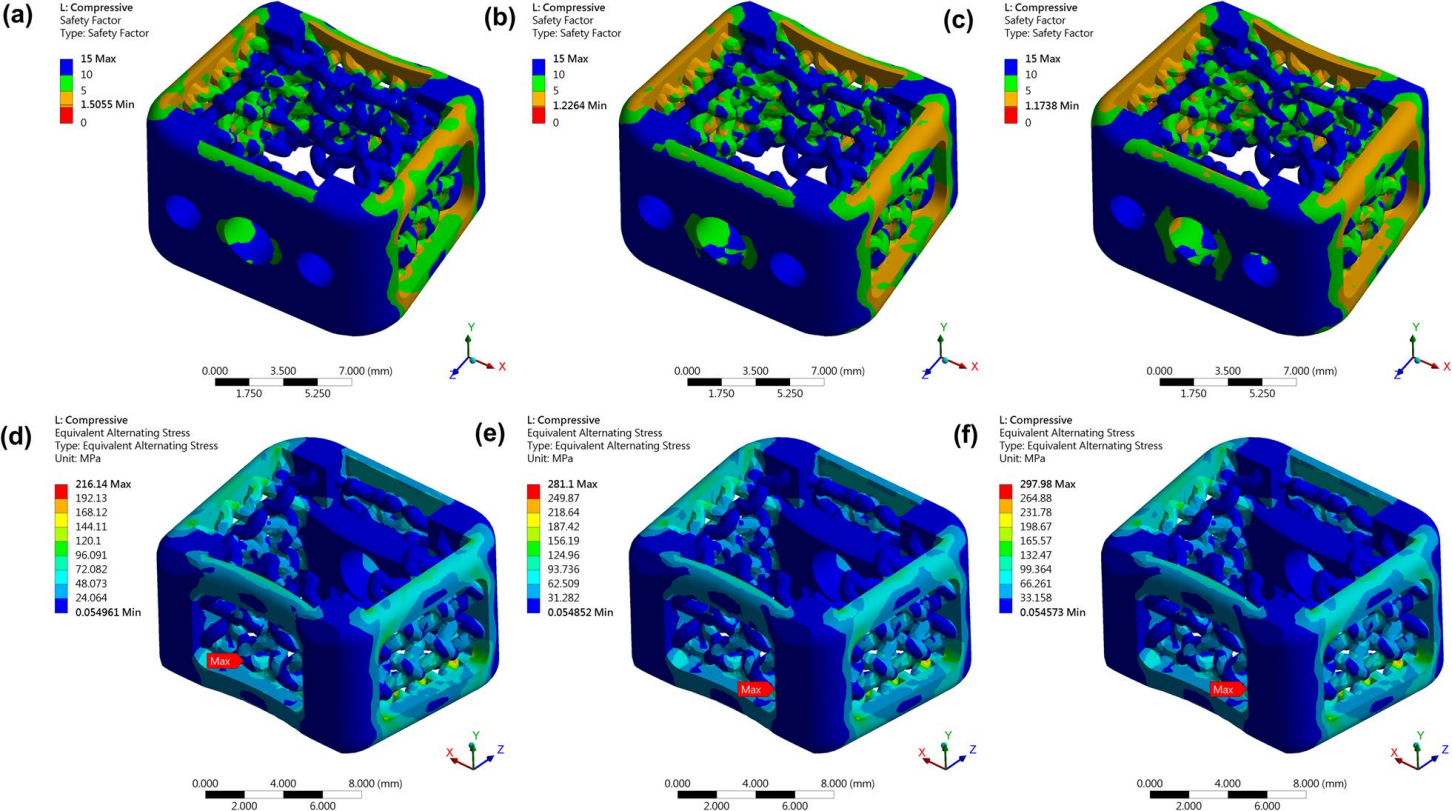
Finite element model: Axial compressive test. **a**, **b** and **c** are alternative stress distributions of cages that were applied 2000N, 2400N, and 2600N respectively. **d**, **e** and **f** are safety factor distributions of cages that were applied 2000N, 2400N, and 2600N respectively

Under the application of 2000N, 2400N, and 2600N axial forces, the minimal safety factor distributions of cages are 1.56, 1.25, and 1.17, respectively [Fig. 3a–c]. These mean that the cages could pass those three dynamic fatigue tests in theoretical situation. Furthermore, to understand weak parts in this cage with axial compressive tests, we plot the alternative stress distribution based on Goodman mean stress theory. The Fig. 3d–f show the distribution of alternative stress distribution, and the location where has a maximum alternative stress is closed to the rear cage. In addition, the stress distribution is located averagely in the front, behind, left, and right regions.

#### Finite element model: torsional test

**Fig. 4:**
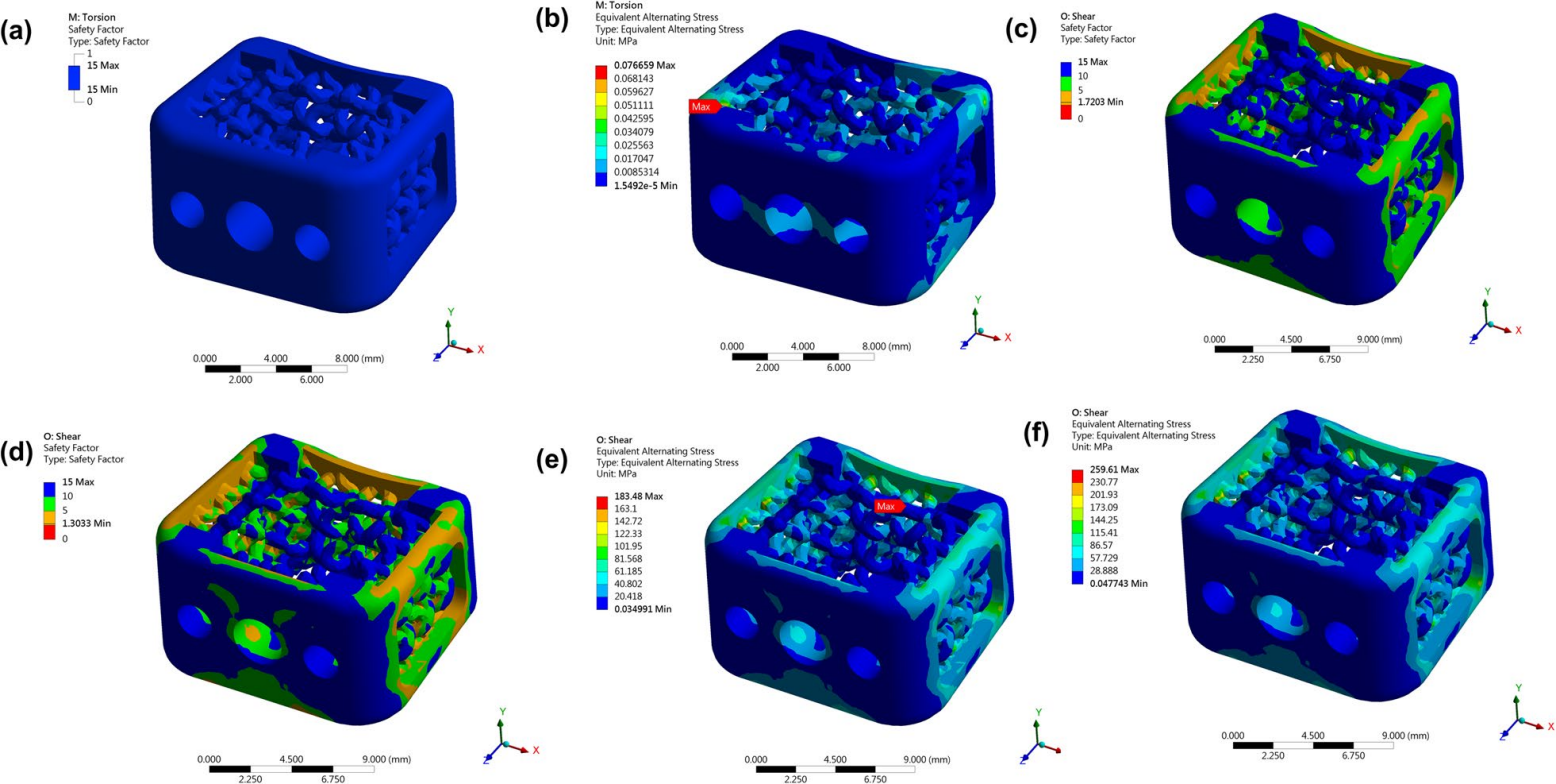
Finite element model: Torsional test, Shear compressive test and Subsidence test. Torsional test: **a** The safety factor distribution for the cage under 2 N-m. **b** The alternative stress distribution for the cage under 2 N-m. Shear compressive test: **c** and **d** are the safety factor distributions for the cages under 1400N and 1875N respectively, and **e** and **f** are the corresponding alternative stress distributions. Maximum plastic stress from quasi-static simulation under 1400N and 1875N respectively

The Fig. 4a shows that the safety factor distribution for the cage under 2 N-m torsion, and the minimum safety factor is 1.5, which is much larger than 1 and means that the cage could successfully pass the loading condition in theory. To further analyze the weak part, we also plot the alternative stress distribution. The maximum alternative stress, illustrated in Fig. 4b, is close to corners of the cage. We can also observe that the stress distribution is more concentrated in the left and right regions. Besides, the maximum plastic strain is 0, which means the cage under the loading condition is still in elastic region.

#### Finite element model: shear compressive test

**Fig. 5:**
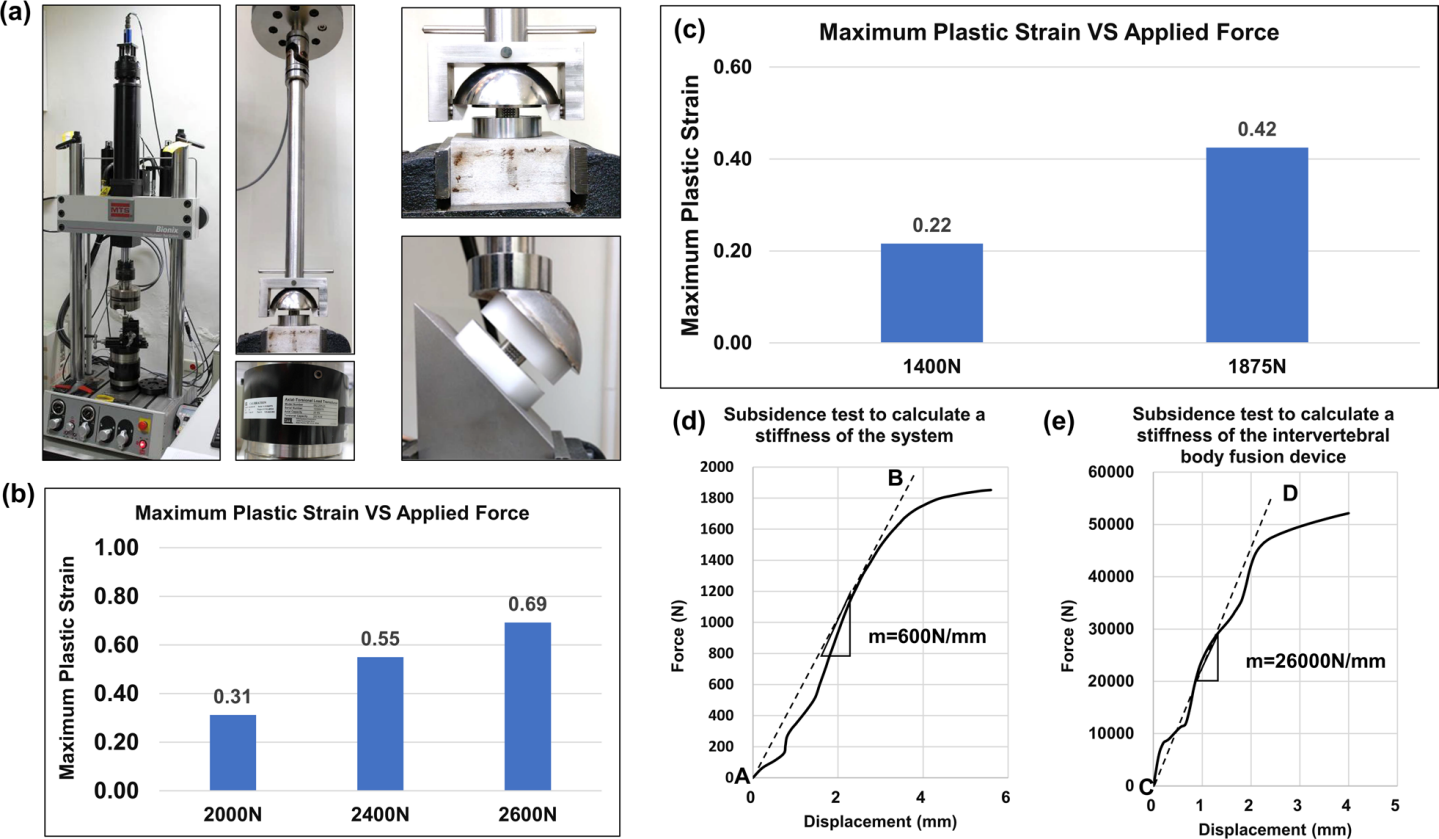
Photo of the setup to perform mechanical tests and biomechanical results of shear compressive test, torsional test, and subsidence tests from quasi-static simulation. **a** By using a material test machine (MTS 370, MTS System Corporation, MN, USA) and MTS torsional load cells (662.18H-05, Axial capacity: 25 KN, Torsional Capacity: 250 N-m), failure of the implants was evaluated. **b** The maximal plastic strain of axial compressive test under 2000N, 2400N, and 2600N respectively. **c** Maximum plastic strain of torsional test under 1400N and 1875N respectively. **d** The loading-displacement graph from the subsidence test for calculating the stiffness of the system. **e** The loading-displacement graph from the subsidence test for calculating the stiffness of the intervertebral body fusion device

The Fig. 4c and d show the distribution of safety factors for the cages under 1400N and 1875N forces. The minimum safety factors are 1.72 and 1.30 respectively. Thus, the cage design could success the shear compressive test. The Fig. 4e and f show the alternative stress distribution, and the values of maximum alternative stress are both located in the upper rear cage. After plotting the maximum plastic strain value versus axial compressive loading from quasi-static simulation, we found that the maximum plastic strain in the stress concentrated region has exceeded 0.3 when the force is 2000N, and the strain exceeded 0.5 and 0.6 when the applied forces are 2400N and 2600N respectively [Fig. 5a]. The maximum plastic strain is 0.21 and 0.42 when the cage was applied 1400N and 1875N respectively [Fig. 5b].

#### Finite element model: subsidence test

The subsidence test focuses on the force–displacement graph and calculates the stiffness of test block, $\mathrm{Kp}$, through Eq. 1. The curve in the beginning is similar to a normal mechanical test, which gradually rise up. However, the curve oscillated after about 0.2 mm, and then the curve steady rise after about 1.8 mm. Regarding the test for calculating the stiffness of system, $\mathrm{Ks}$, the result is illustrated in Fig. 5c and shows that the stiffness of the system,$\mathrm{Ks}$, is 600 N/mm. After calculating the stiffness of test block, $\mathrm{Kp}$, by using the Eq. (1), the value is 617 N/mm. Beside, we also found that the behavior of the test for calculating the $\mathrm{Ks}$ seems reversed. Compared to the curve from the test for calculating the $\mathrm{Kd}$, the curve in Fig. 5c oscillated in the beginning and gradually became consistent. The Fig. 5d, e shows the loading-displacement plot of the test to calculate, $\mathrm{Kd}$. The slope of the line AB is the stiffness of the cage, $\mathrm{Kd}$, which is 26000N/mm.

### Biomechanical test results

**Fig. 6:**
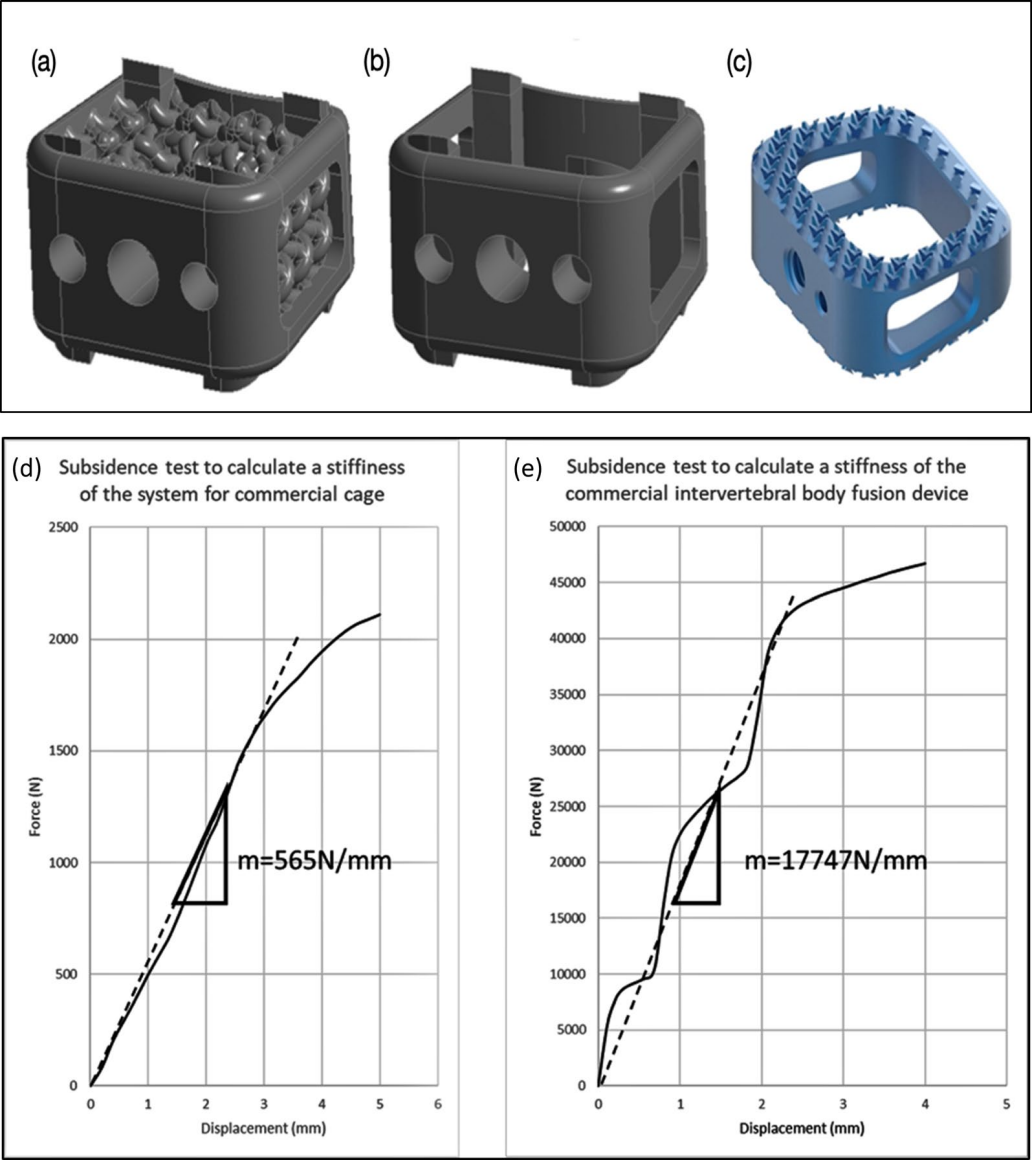
Comparison with the conventional design. Upper: The designs of **a** the proposed biomimetic cage, **b** the control cage based on **c** a typical conventional cage with outer frames only. Lower: **d** The loading-displacement graph from the subsidence test for calculating the stiffness of the system of the control case. **e** The loading-displacement graph from the subsidence test for calculating the stiffness of the control case of the intervertebral body fusion device

Table 2 lists the result for each test parameter. It is important to note that the ranges of yield and ultimate strengths reported in Table 2 represent a subset of devices for which the behavior was analyzed with or without hot isostatic pressing (HIP) treatment. After HIP treatment, the tensile test specimen has higher stretching rate, lower tensile strength, but the yielding strength in stretching test was significantly higher (Additional file 1: Table S2). The tensile test specimen failure mode usually resulted in fracture, which specification and failure mode were shown in Fig. 6. The most common failure mode for all static tests was plastic deformation or destruction of the device. Axial compression failure modes typically usually resulted in buckling of the portion of the device, cracks propagating from features such as side windows, or device endplates. Shear failures often involved crack propagation and plastic deformation of the device in the direction of shear force was applied. Torsion failures most commonly resulted in crack propagation and plastic deformation of the device in the direction of torsion was applied (Fig. 6). The subsidence testing consisted of the device sinking into the polyurethane foam test block, without structural failures of the cervical interbody fusion devices. In certain instances, cracks propagated to the point of complete device fracture into two or more pieces (Fig. 6). Slippage of the device from the test blocks during static compression-shear and static torsion testing were also noted.

### Validation of the finite element analysis

**Fig. 7:**
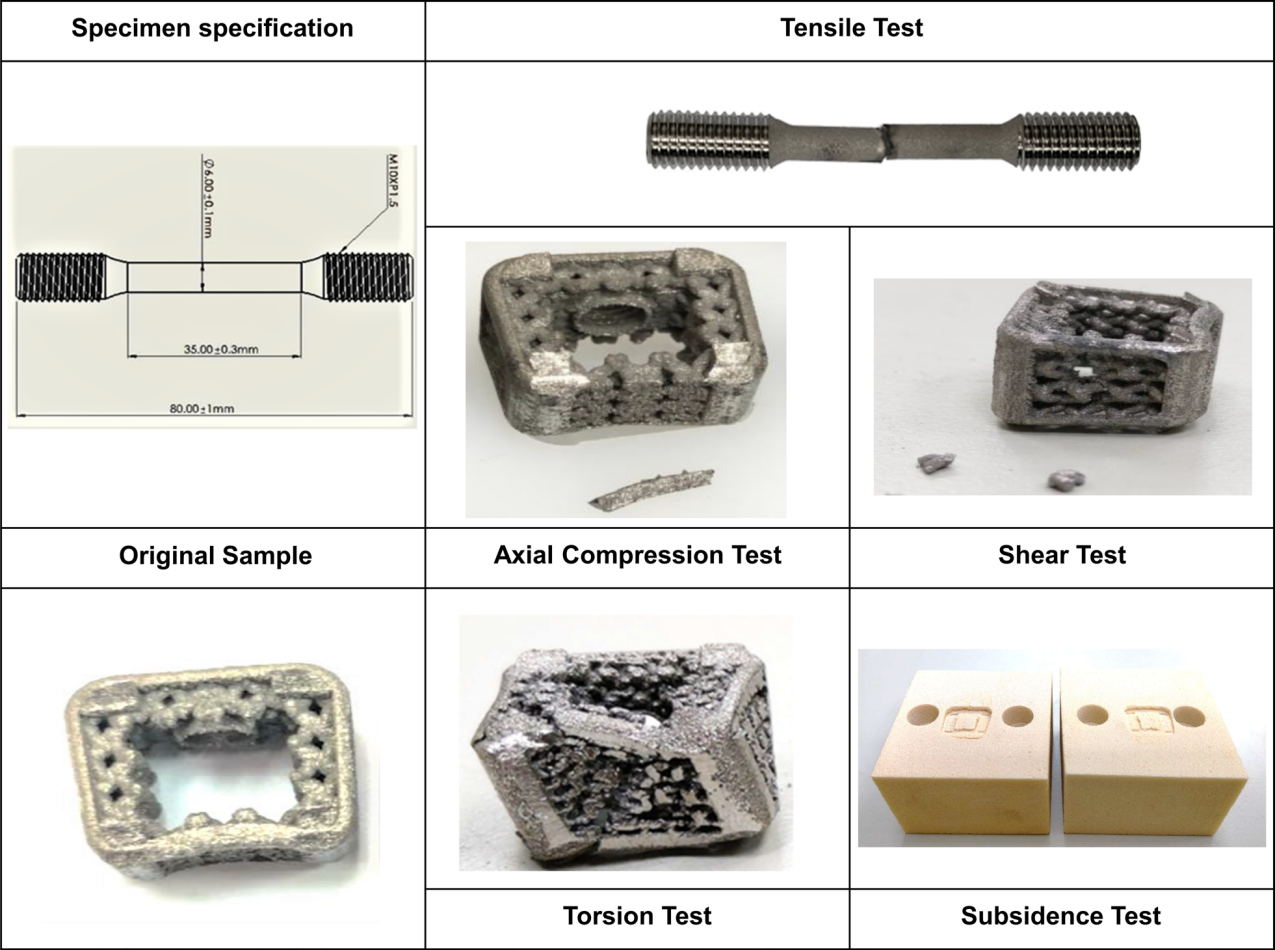
Failure modes of different mechanical tests. Static axial compression usually resulted in buckling of the portion of the device not shielded by the fixture blocks. Static compression-shear usually resulted in plastic deformation of the device in the direction shear force was applied. The failure mode during subsidence testing consisted of the device sinking into the polyurethane foam test block, with no structural failures of the cervical interbody fusion devices reported

The results generated by the current finite element models can be validated by correlating the location of the maximum stress in the models and the failure location of the cage found in the experiments. For example, the result of axial compressive simulation shows that the maximum stress occurred at the distal side of the cage, which has good agreement with the failure location found in the experimental result, as shown in Fig. 7a. The result of shear compressive simulation shows that the maximum stress occurred at the distal part and diagonal opposite posterior part of the cage, showing good agreement in the experimental result, as shown in Fig. 7b. The results of torsion simulation and experimental tests both show that the maximum stress occurred at the corner of posterior part of the cage, as shown in Fig. 7c. Thus, we can conclude the current finite element models can produce reasonable results and are well correlated to the experimental approaches.

### Comparison with the conventional design

**Fig. 8:**
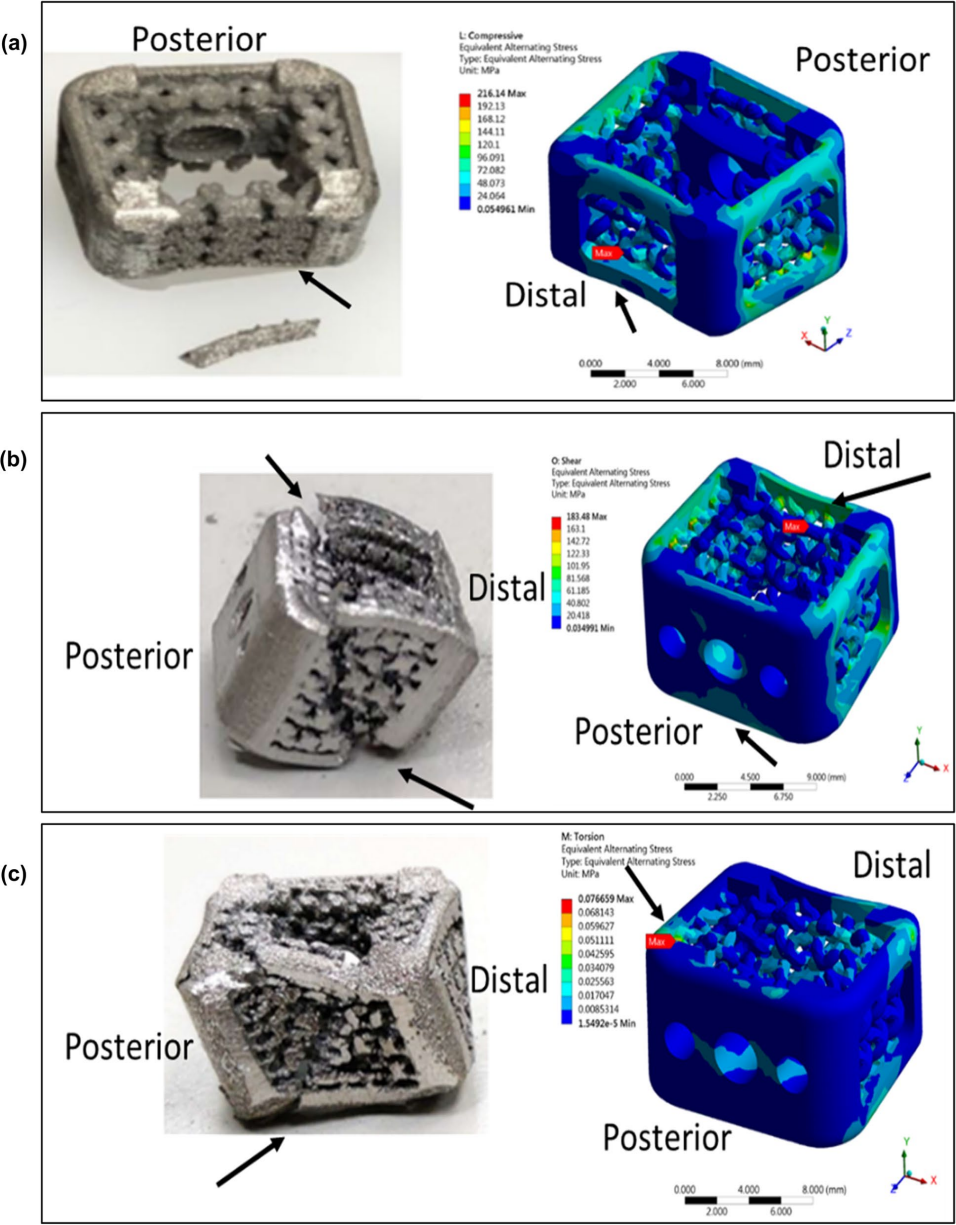
Validation of the finite element analysis: Similar failure locations at both finite element (FE) and experiment of the cage. **a** Both FE and experiment results in the axial compressive tests show similar failure locations around distal part of the cage. **b** Both FE and experiment results in the shear compressive tests show similar failure locations around distal part and diagonal opposite posterior part of the cage. **c** Both FE and experiment results in the torsional tests show similar failure locations around the corner of posterior part of the cage

In order to demonstrate the good subsidence performance of the proposed cage [Fig. 8a], a similar cage without the biomimetic design was also built as a control case [Fig. 8b]. The design is based on a typical conventional cage with outer frames only [Fig. 8c]. A subsidence test simulation was performed for the control cage. The value of *K*_s_ and *K*_d_ are 565 and 17747 N/mm, respectively, as shown in Fig. 8d, e. Thus, the corresponding *K*_p_ of the control cage is 584 N/mm. We can conclude that the biomimetic design provides better performance (*K*_p_ = 617 N/mm) in the subsidence test simulation by about 5% compared with the cage based on the conventional design.

**Fig. 9:**
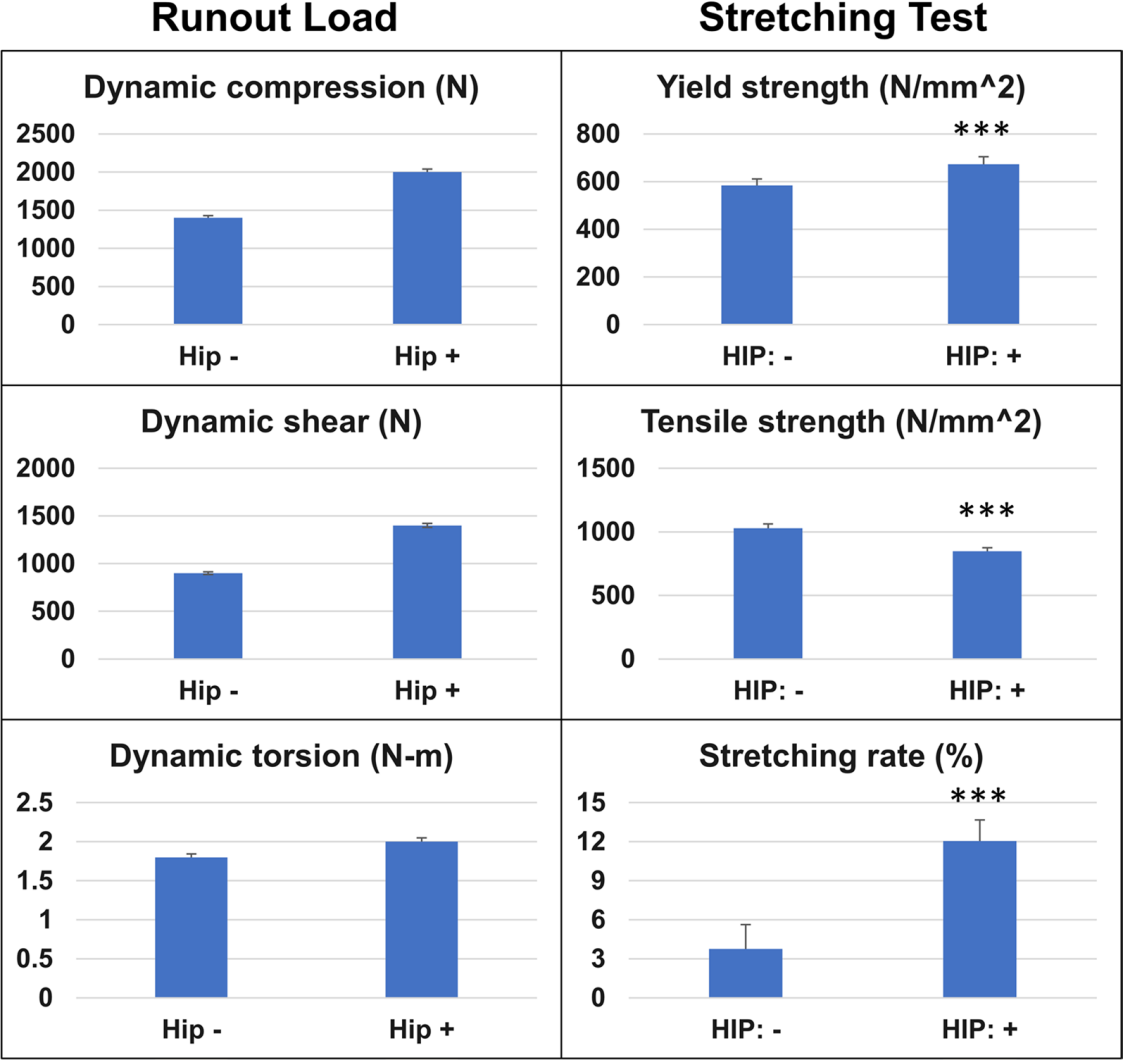
Mechanical properties before and after hot isostatic pressing (HIP) treatment. After hot isostatic pressing (HIP) treatment, there were significant changes of mechanical properties. In the dynamic tests, the runout load of compression, shear and torsion tests were all increased; while for the stretching test, the tensile strength was significantly decreased, and the yield strength and stretching rate were significantly increased

According to ASTM F1839, sawbones used in the subsidence performance test, the results showed the block stiffness (Kp) was 436.3 ± 20.98 N/mm (Additional file 1: Table S3). Dynamic tests for fatigue were also performed for the devices before and after hot isostatic pressing (HIP) (Table 1). After hot isostatic pressing (HIP) treatment, there were significant changes of mechanical properties in tensile test (Fig. 9). In the dynamic tests, the runout load of compression, shear and torsion tests were all increased. For the tensile test, the tensile strength was significantly decreased, while the yield strength and stretching rate were significantly increased.

**Table 1:** Dynamic tests for fatigue

Torsion (N-m)	% of static strength	HIP−		HIP +	
		Result (cycle)	Failure mode	Result (cycle)	Failure mode
*Dynamic torsional test for fatigue*					
− 10 ~ 10	25.00	Failure (16,042)	Fracture	N/A	N/A
− 5 ~ 5	12.50	Failure (635,681)	Fracture	N/A	N/A
− 2.5 ~ 2.5	6.30	Failure (2,096,610)	Fracture	N/A	N/A
− 2 ~ 2	5.00	Surviving (4,105,587)	Fracture	Surviving (5,000,000)	No failure
− 1.8 ~ 1.8	4.50	Surviving (5,000,000)	No failure	N/A	N/A

Axial load (N)	% of static strength	Result (cycle)	Failure mode	Result (cycle)	Failure mode
*Dynamic Axial Compression Test for Fatigue*					
300 ~ 3000	11.40	Failure (960,322)	Fracture	N/A	N/A
220 ~ 2200	8.40	Failure (19,258,030)	Fracture	Surviving (5,000,000)	No failure
160 ~ 1600	6.10	Failure (3,255,271)	Fracture	N/A	N/A
140 ~ 1400	5.30	Surviving (5,000,000)	No failure	N/A	N/A
*Dynamic Shear Test for Fatigue*					
200 ~ 2000	27.40	Failure (91,358)	Fracture	N/A	N/A
140 ~ 1400	19.20	Failure (204,641)	Fracture	Surviving (5,000,000)	No failure
100 ~ 1000	13.70	Failure (3,910,002)	Fracture	N/A	N/A
80 ~ 800	11.00	Surviving (5,000,000)	No failure	N/A	N/A
90 ~ 900	12.30	Surviving (5,000,000)	No failure	N/A	N/A

HIP treatment − (without hot isostatic pressing); HIP treatment: + (with hot isostatic pressing)

### Comparison with FDA post-market medical device regulation (MDR) databank

The ten most reported events associated with these MDRs from FDA databank [3] were compared with our device (Table 2). For the static mechanical tests, the results were all located at 75th to 95th percentile; while for the dynamic tests, runout load for axial compression was located at 25th to 50th percentile, compression shear at 50th-75^th^ percentile, and the torsion rounout torque at 75th to 95th percentile, and the subsidence was located at the 50^th^ to 75^th^ percentile (Table 2).

**Table 2:** Comparison of mechanical testing results with FDA mechanical testing data bank

	Test	Test parameter	5th percentile	25th percentile	50th percentile	75th percentile	95th percentile	Present study Mean ± S.D
Static (ASTM F2077)	Axial compression	Stiffness (N/mm)	5097	7984	10,108	13,300	19,203	26,244.8 ± 1154.0
		Yield strength (N)	5450	8379	10,117	12,131	15,256	
		Ultimate strength (N)	6236	8935	10,800	14,728	32,863	26,322.1 ± 1848.5
	Compression—shear	Stiffness (N/mm)	1492	2927	4347	6140	10,538	9514.0 ± 307.6
		Yield strength (N)	1464	2447	3680	5265	6685	7230.8 ± 332.5
		Ultimate strength (N)	1515	2861	4626	6868	11,001	7334.8 ± 379.5
	Torsion	Torsional stiffness (N m/degree)	0.3	0.7	1	1.9	4.7	5.5 ± 0.3
		Yield moment (N m)	3.1	6.1	8.6	12	18.8	
		Ultimate moment (N m)	3.3	7.6	9.9	13.8	25.3	39.7 ± 1.5
Dynamic (ASTM F2077)	Axial compression	Runout load (N)	1500	2000	2600	3500	5760	2000–2400
	Compression—shear	Runout load (N)	679	1000	1400	1875	2450	1400–1875
	Torsion	Runout torque (N m)	± 1.0	± 1.5	± 1.5	± 2.0	± 3.0	> 2
Subsidence (ASTM F2267)		Kp, Block stiffness (N/mm)	257	324	424	522	791	436.3 ± 21.0

The FDA Mechanical testing results was cited from Peck JH et al. [3]

## Discussion

Anterior cervical discectomy and fusion (ACDF) has been a standard intervention for cervical intervertebral disc degenerative diseases. The ideal implant should be biocompatible and have adequate physical and mechanical properties. To maintain or restore disc height while providing stability to allow for the development of a fusion mass, these intervertebral body fusion devices (IBFDs) need to withstand the physiologic loading in the cervical spine while avoiding significant subsidence into the vertebral bodies [30]. ASTM F2077 Test Methods for Intervertebral Body Fusion Devices contains methods for performing static and dynamic axial compression, compression-shear, and torsion testing on IBFDs, these methods allow for mechanical properties to be compared between devices under the predominant loading modes an IBFD is expected to experience in vivo; while ASTM F2267 Standard Test Method for Measuring Load Induced Subsidence of Intervertebral Body Fusion Device Under Static Axial Compression contains methods to test the propensity of an IBFD to subside [3]. These standards describe methods were used in this study to evaluate the mechanical properties of our 3D printed biomimetic porous device.

Our mechanical simulation of dynamic fatigue tests showed that the cage could success at most 2600N for axial compressive test, 2N-m for torsional test, and 1875N for shear compressive test. However, compared with the real mechanical experiments, our cage only success the 2000N for axial compressive test, 2N-m for torsional test, and 1400N for shear compressive test. Considering the values of safety factor, the Goodman mean stress theory might be less accurate for the higher plastic strain. It seems that a cage could succeed in a test when a value of safety factor is larger than 1.3. In the shear compressive test, even if the cage has 1.3 safety factor, it is still failed in the real experiment. As a result, we couldn't only use whether a value of safety factor is larger than 1 as the index to evaluate the design because the Goodman mean stress theory would have less accuracy when plastic strain occurred.

**Fig. 10:**
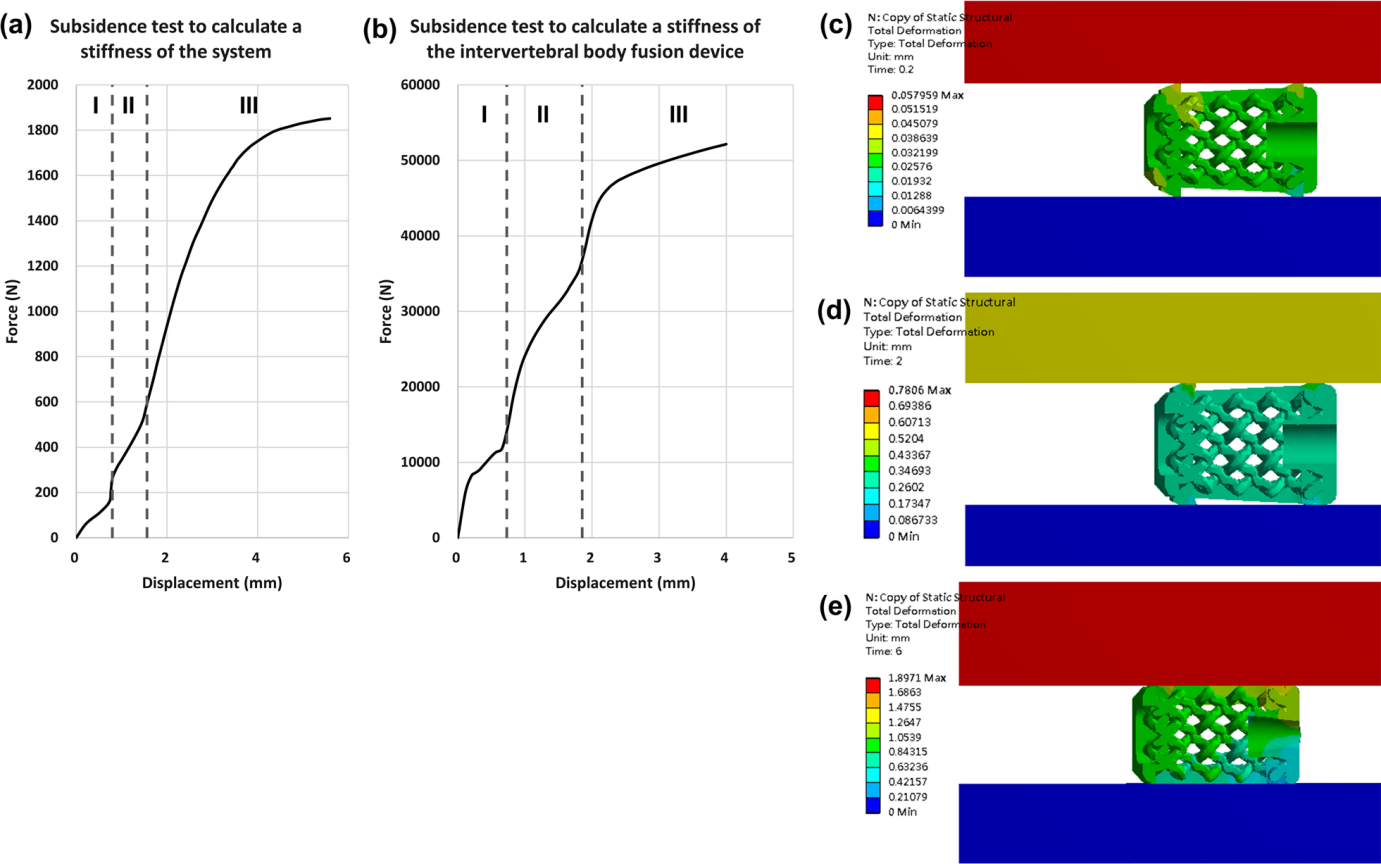
Turning points of the subsidence test curve. **a** and **b** are the curve from subsidence test, which are separated into three regions. **c** The total deformation of the cage for subsidence test in region I. **d** The total deformation of the cage for subsidence test in end of region I. **e** The total deformation of the cage for subsidence test in end of region II

Regarding the subsidence test, we can observe a few turning points in the Fig. 10a and b, and the points are resulted from the design of cage. We can separate the curve into three regions, which are represented by region I, region II, region III as shown in Fig. 10a and b. The region I is contributed by the design that is used to anchor the cage into cervical spine end plate. The sharp and obtrusive parts initially contacted the structural steel test blocks, and those parts are still in elastic zone. After the obtrusive parts were severely compressed and exceed their local yielding point, the slope gradually decreased until the test blocks contacted the front cage with larger height and entered region II. The width of the region I have the same value as the height of the front obtrusive parts. In addition, Fig. 10c is the total deformation of the test in the end of region I and showed that the test blocks contacted the front cage. Then, the slope of the curve increased in region II in the beginning because the front cage is still in elastic zone and has higher stiffness. However, the slope decreased again because the front cage exceeded its local yielding point, and the total deformation of the test in the end of region II is showed in Fig. 10**(d)**. When the test entered the region III, the slope increased again because the test block contacted the rear cage, and the blocks fully contacted the whole cage as showed in Fig. 10e. In the end, the rear part exceeded its local yielding point, and the slope of the curve gradually decreased. The whole process could be also observed in the test for calculating the stiffness of the system, $\mathrm{Ks}$, even though it's not so obvious. To design a cage with higher stiffness of test blocks, $\mathrm{Kp}$, we could not only increase the contact area but also decrease the length of those obtrusive parts. According to the Eq. (1) and our simulation results, the region II and III contribute most of the $\mathrm{Kp}$, and the deformation occurred in region I make the $\mathrm{Kp}$ decrease. Thus, apart from increasing the contact area to increase the stiffness contributed by region II and III, we could also decrease the height of obtrusive parts, which could decrease the width of region I and increase $\mathrm{Kp}$.

The structural support of the interbody device can provide biomechanical stability, while consistent loading of the bone graft material can accelerate mechanotransduction and bone remodeling; both factors lead to a successful intervertebral fusion. High stiffness of the cage may cause stress concentration and a stress shielding effect between the vertebral bones and the cages; then the stress shielding effect easily causes damage and leading to a higher risk of reoperation [31]. Design strategies such as contact area, open architecture (i.e., pores) to allow for multidirectional bone ingrowth, conformity, and direct loading of the graft material had been shown to accelerate bone healing and interbody fusion formation [32]. A porous structure for the spinal fusion device can effectively reduce the stiffness to obtain more comparative strength for the surrounding tissue, this results in uniform distribution of the stress and strain of the devices with the human bone and reduce the stress concentration [31]. With the help of selective laser melting (SLM) technology, Additive manufacturing technology is possible to quickly produce fully functional and complexly-shaped parts, which are often not produced by other conventional technologies. Nowadays, 3D printing products are broadly used in a wide range of applications, for the medical industry where they are most often used as hard tissue replacements [33]. In a prospective cohort reporting the clinical and quantitative radiological outcome of 3-D printed porous titanium implants; the 3-D printed porous titanium cervical implants had a significant better results in clinical improvement after surgery, faster bony consolidation and one level anterior cervical fusion successfully achieved without additional plating, and the fusion rate was similar to that with autograft [34].

The fabrication of material via 3-D printing technology has been developed in the past decade due to the convenience of the complex geometry design, faster manufacturing times and fine microstructure. However, several problems such as porosity, residual internal stresses and anisotropic properties, make it necessary to heat treat the components to modify their microstructure and improve their performance. A series of post heat treatments is required to reduce internal stresses, increase density and develop the final shape, finish and (most importantly) microstructural phases, resulting in the desired physical properties. After stress relief, components may be required to go through the hot isostatic pressing (HIP) process to eliminate pores and heal defects, achieving 100% of the maximum theoretical density [33]. Hot isostatic pressing (HIP) is a form of heat treatment that uses high pressure to improve material properties. This post heat treatment leads to the loss of the initial fine microstructure and hence the high strength [35]. In this study, Ti-6Al-4 V coupons were manufactured using selective laser melting (SLM) and subsequently post-processed using combinations of hot isostatic pressing (HIP). The temperature applied was 890 °C (holding time: 120 min; heating rate: 0.5 °C min^−1^), it can reduce residual stress without significant microstructural changes. There was also no phase transformation resulting in mechanical stress accumulation.

Cage subsidence is one of the major complications after spinal fusion. Various aspects of cage design have been investigated for their influence on cage subsidence, whereas graft material plays a role in reducing peak endplate pressures, implant subsidence, as well as bone remodeling [36]. The biomechanical properties of the implant are essential to its stability [11]. A previous study reported that metal implants with high stiffness could cause stress shielding of the bone surrounding the prosthesis, thereby limiting the load transferred to the bone [37]. Therefore, titanium alloys share part of the load previously withheld merely by bones. According to Wolfe’s law, the structure of the bones is suitable for resisting any force acting on the bones [12], and the bone mass is reduced in response to low stress. Thus, the mismatch in stiffness between the Ti-6Al-4 V and bone can lead to stress shielding, resulting in bone resorption and implant loosening. 3D-printed porous titanium cage can minimize stress-shielding [9], restores the surgical segmental curvature, maintains the intervertebral height, and prevents postoperative subsidence-related complications [38]. The optimized interbody fusion device, we further reduce the stiffness through structural optimization and provides the bone graft greater mechanical stimulation while ensuring stability.

A structure with lower stiffness should theoretically maintain a certain degree of movement and reduce the stress on the facet joints and intervertebral discs in adjacent positions [13]. It was measured by Grant et al. that the stiffness of different areas on the endplate exhibited a trend of decreasing from the outside to the center of the endplate [14]. Microfractures occur when the local stress is higher than the limit of the relevant area [14, 15], leading to osteolysis and cage subsidence [14, 15]. A previous study reported that micro-movements greater than 150 μm reduced the interface bonding strength, eventually resulting in implant relaxation [16]. Our biomimetic porous devices exhibited that the outer pore size was around 500–600 μm to facilitate the vascular ingrowth; however, the average block stiffness of subsidence was with the 50^th^ to 75^th^ percentile, while the axial compression stiffness was 95^th^ percentiles, the shear stiffness and torsional stiffness were around 75^th^ to 95 percentiles of current devices. Therefore, the total strength of the overall cage system to resistant compression or shear or rotation stress was within the safe range, which resulted in no significant change in the adjacent intervertebral disc force. We believe that the lower the stiffness of the implant, the smaller the stress on the endplate, the lower possibility of occurrence of microfractures, osteolysis, or cage subsidence, while the inner pore size decrease gradually in this design to enhance the bone ingrowth. However, the bone-cage interface in the surgery models was too simplified and further studies are still required to investigate these variables.

## Conclusions

With the help of selective laser melting (SLM) technology, additive manufacturing 3-D printing technology was used to produce a biomimetic porous interbody fusion devices. A post heat treatment was used to optimize its mechanical properties, as the stiffness of the device decreases to reduce the stress shielding effect between two instrumented bodies. In summary, we’ve successfully designed a porous cage based on the biomechanical load through lattice optimization. This optimized device with reduced stiffness can decrease the stress shielding effect, and provide appropriate space for bone growth.

## Supplementary Information

**Additional file 1. Supplementary-Table 1**. The boundary conditions and material properties used in the mechanical simulation.** Supplementary-Table 2**. Mechanical testing results.** Supplementary-Table 3**. Subsidence performance for implants before hot isostatic pressing (HIP). (n=5).

## Data Availability

The datasets generated during and analyzed during the current study are not publicly available due to some data of project is not suitable for disclosure but are available from the corresponding author on reasonable request.
