# Supplementary material for: Mechanical performance of porous biomimetic intervertebral body fusion devices: an in vitro biomechanical study
Source: J Orthop Surg Res. 2023 Jan 30;18:71. doi: 10.1186/s13018-023-03556-4 (PMC9885572; doi:10.1186/s13018-023-03556-4)
Supplement: Supplementary file 1 — Additional file 1. Supplementary-Table 1. The boundary conditions and material properties used in the mechanical simulation. Supplementary-Table 2. Mechanical testing results. Supplementary-Table 3. Subsidence performance for implants before hot isostatic pressing (HIP). (n=5). [file 13018_2023_3556_MOESM1_ESM.docx]

**Supplementary-Table. 1**

The boundary conditions and material properties used in the mechanical simulation.

| Test Type | Boundary condition |  | Component | | Material | Elastic modulus | Poisson's ratio | Yielding stress | | |  |
| --- | --- | --- | --- | --- | --- | --- | --- | --- | --- | --- | --- |
| Axial compressive test | 2000N |  | Test Block for fatigue test | | Polyacetal | 3.1GPa | 0.25 | 72 MPa | | |  |
|  | 2400N |  | Fixture | | Structural Steel | 210GPa | 0.3 | NA | | |  |
|  | 2600N |  | Cage | | Titanium Alloy | 108.7GPa | 0.36 | 849.7 MPa | | |  |
| Torsional test | 1400N |  | Test Block for subsidence test | | Grade 15 polyurethane | 123 MPa | 0.3 | 4.9 MPa | | |  |
|  | 1875N |  |  | |  |  | | |  | |  |
| Shear compressive test | 2N-m |  | |  |  |  | | | |  |  |

**Supplementary-Table 2**

Mechanical testing results.

|  | **HIP: -**  Mean±S.D. | **HIP: +**  Mean±S.D. | P Value |
| --- | --- | --- | --- |
| **Stretching test (n=12)**  **ASTM E8/E8M-2016a.** |  |  |  |
| Yield strength (N/mm^2) | 584.2±27.3 | 673.0±31.4 | 1.4813E-17 |
| Tensile strength (N/mm^2) | 1027.9±34.8 | 846.8±28.7 | 3.1846E-19 |
| Stretching rate (%) | 3.8±1.9 | 12.0±1.6 | 4.5737E-12 |
| **Torsional test (n=5)**  **ASTM F-2077-18** |  |  |  |
| Torsional Stiffness (N-m/deg) | 5.5±0.3 | N/A |  |
| Max. Torque (N-m) | 39.7±1.5 | N/A |  |
| Displacement at Max. Torque (deg) | 14.7±0.8 | N/A |  |
| **Axial Compression Test (n=5)**  **ASTM F-2077-18** |  |  |  |
| Axial Stiffness (N/mm) | 26244.8±1154.0 | N/A |  |
| Max. Load (N) | 26322.1±1848.5 | N/A |  |
| Displacement at Max. Load (mm) | 2.0±0.0 | N/A |  |
| **Shear Test (n=5)**  **ASTM F-2077-18** |  |  |  |
| Yield Load (N) | 7230.8±332.5 | N/A |  |
| Yield Displacement (mm) | 1.4±0.05 | N/A |  |
| Stiffness (N/mm) | 9514.0±307.6 | N/A |  |
| Max. Load (N) | 7334.8±379.5 | N/A |  |
| Displacement at Max. Load (mm) | 1.4±0.1 | N/A |  |

HIP treatment: - (without hot isostatic pressing); HIP treatment: + (with hot isostatic pressing)

**Supplementary-Table 3**

Subsidence performance for implants before hot isostatic pressing (HIP). (n=5)

| Subsidence performance Test  ASTM F-2067-04(2018) | Mean ± S.D. |
| --- | --- |
| Kd (N/mm) | 26245.0±1153.88 |
| Ks (N/mm) | 429.1±20.55 |
| Kp (N/mm) | 436.3±20.98 |

According to ASTM F1839, sawbones used in this study was polyurethane test block (Polyurethane test block, product number: 1522-02, Pacific Research Laboratories Inc., U.S.).

Kp= (Ks*Kd)/ (Kd-Ks)

Ks: system stiffness; Kp: polyurethane test block; Kd: specimen stiffness .
